# Do routine outcome monitoring results translate to clinical practice? A cross-sectional study in patients with a psychotic disorder

**DOI:** 10.1186/s12888-016-0817-6

**Published:** 2016-04-18

**Authors:** Magda Tasma, Marte Swart, Gert Wolters, Edith Liemburg, Richard Bruggeman, Henderikus Knegtering, Stynke Castelein

**Affiliations:** Lentis Research, Lentis Psychiatric Institute, Groningen, The Netherlands; Rob Giel Research Center, University of Groningen, University Medical Center Groningen, Groningen, The Netherlands

**Keywords:** Mental health care, Routine outcome monitoring, Implementation, Clinical practice, Psychotic disorders, Positive symptoms, Negative symptoms, Psychosocial problems, Cardiovascular risk factors, Non-detection

## Abstract

**Background:**

The use of Routine Outcome Monitoring (ROM) in mental health care has increased widely during the past decade. Little is known, however, on the implementation and applicability of ROM outcome in daily clinical practice. In the Netherlands, an extensive ROM-protocol for patients with psychotic disorders has been implemented over the last years (ROM-Phamous). The current study investigated to what extent ROM results translate to daily clinical practice. Therefore, we investigated whether clinical problems as identified with ROM were detected and used in the treatment of patients with psychotic disorders.

**Methods:**

Out of the ROM database of 2010 (*n* = 1040), a random sample of 100 patients diagnosed with a psychotic disorder was drawn. ROM-data used in this study included a physical examination, laboratory tests, interviews and self-report questionnaires. Based on these data, the prevalence of positive and negative symptoms, psychosocial problems and cardiovascular risk factors was determined. Next, we investigated whether these problems, as identified with ROM, were reflected in the treatment plans of patients, as an indication of the use of ROM in clinical practice.

**Results:**

The sample consisted of 63 males and 37 females. The mean age was 44 and the mean duration of illness was 17.7 years. The prevalence of positive and negative symptoms, psychosocial problems and cardiovascular risk factors ranged from 11 to 86 %. In the majority of cases, problems as identified with ROM were not reflected in the treatment plans of patients.

**Conclusions:**

We found a substantial discrepancy between the ROM measurements and the treatment plans, i.e. low rates of detection of symptoms, psychosocial problems and cardiovascular risk factors in the treatment plans, even though these problems were identified with ROM. The opposite occurred as well, where problems were reflected in the treatment plans but not identified with ROM. Thus, ROM and daily clinical practice appear to be two separate processes, whereas ideally they should be integrated. Strong efforts should be made to integrate ROM and consequent treatment activities. Such integration may help to provide patients with adequate and customized care and simultaneously minimize under- and over-treatment.

## Background

Psychiatric disorders with a psychotic component include schizophrenia, schizophreniform disorder, schizoaffective disorder, delusional disorder, brief psychotic disorder, psychotic disorder not otherwise specified, and psychosis associated with substance use or medical conditions, according to the DSM-IV-TR classification system [1]. Core features of most psychotic disorders are positive symptoms (hallucinations, illusions, delusions, incoherent thoughts and problems with logical thinking) and negative symptoms (loss of initiative, flat affect, poverty of speech, lack of motivation and social withdrawal) [1, 2]. The prevalence of schizophrenia worldwide is in the range of 1.4 to 4.6 per 1000 people, pending a large burden on patients and society [2, 3].

Both positive symptoms and negative symptoms greatly interfere with daily life functioning [2]. Moreover, patients have reported daytime activities, psychotic symptoms, psychological distress, company and intimate relationships as the most frequently occurring unmet needs [4]. As a result, patients with psychotic disorders often experience reduced quality of life [5]. Furthermore, life expectancy is significantly lower (20–25 years) [6–9] and mortality rates are 2–3 times greater than in the general population [10]. Especially premature cardiovascular disease has been associated with lower life expectancy and high mortality rates [11]. In patients with psychotic disorders, risk factors for cardiovascular disease are often present, such as obesity (45–55 %), smoking (50–80 %), diabetes mellitus (10–15 %), hypertension (19–58 %) and dyslipidaemia (25–69 %) [12]. Of note, these medical problems often go undetected or untreated in patients with severe mental illness [13].

Routine Outcome Monitoring (ROM) in adult patients may improve the diagnostics and treatment of psychiatric problems, according to a review of Carlier et al. [14]. However, several other studies claim that few clinicians in psychiatry use the outcome of ROM in their day-to-day work [15, 16]. During the last decade, the use of ROM in mental health care has increased widely. This increase is partly due to demands of health insurance companies [17]. Little is known, however, on the implementation of ROM-measurements and the applicability of its outcome in daily clinical practice.

Over the last years, a protocol called the Routine Outcome Monitoring Pharmacotherapy Monitoring and Outcome Survey (ROM-Phamous) has been developed in the Northern Netherlands (Bartels-Velthuis A, Visser E, Arends J, Pijnenborg G, Wunderink L, Jörg F, et al: Pharmacotherapy Monitoring and Outcome Survey: the annual PHAMOUS screening of physical and mental health of patients with psychotic disorders, in preparation) [18], to optimize psychosocial interventions and pharmacotherapy. This protocol prescribes a yearly assessment with a comprehensive battery of instruments to monitor psychiatric symptom severity, medication-efficacy and physical health, social functioning, quality of life and service user satisfaction in all patients with psychotic disorders.

The present study investigated to what extent ROM results of patients with a psychotic disorder translate to daily clinical practice. To this end, we investigated whether clinical problems as identified with ROM were reflected in the treatment plans of patients. The frequently occurring clinical problems that we chose to investigate, were positive and negative symptoms, problems with social functioning, problems with daily activities and cardiovascular risk factors. As previous studies have shown that clinicians do not always use ROM outcome in their day-to-day work [15, 16] and various problems in patients with a psychotic disorder remain undetected or untreated [13], we expect a discrepancy between problems identified with ROM and the problems that are reflected in the treatment plans of patients with a psychotic disorder.

## Methods

### Subjects

In 2010, the ROM-Phamous database contained information of 1040 patients who received care at Lentis Psychiatric Institute or at the University Center of Psychiatry of the University Medical Center Groningen (UMCG) in the Netherlands. The current study could be performed without extra assessments of the patients, since the ROM-data were available at the start of the study. The Medical Ethical Committee of the UMCG has confirmed that anonymized ROM-Phamous data may be used for scientific research and, therefore, this study did not require additional ethics approval. The study was executed in line with local legislation and the Declaration of Helsinki.

### Procedure

The ROM-Phamous screening protocol is extensive and contains a battery of instruments, including a physical examination (weight, height, abdominal circumference, blood pressure, movement tests), laboratory tests (including glucose and cholesterol levels), an interview (PANSS [19]), and clinician-rated and self-report questionnaires (HoNOS [20], MANSA [21], SRA-34 [22]). In our cross-sectional study, the prevalence of positive and negative symptoms, psychosocial problems and cardiovascular risk factors was calculated with the available ROM-data. Next, the psychiatric treatment plans, obtained from the Electronic Patient File (EPF), were investigated. The first treatment plan written after the ROM-screening was used for the study. Treatment plans of patients receiving care at Lentis Psychiatric Institute were retrieved by a clinician of this institute (GW). Treatment plans of patients receiving care at the University Medical Center Groningen were retrieved by a clinician of this hospital (RB). The heads of the departments at Lentis Psychiatric Institute and the University Medical Center Groningen provided permission to access the treatment plans for this study. All treatment plans were stored anonymously and investigated by an independent researcher (MT). The investigation of the treatment plans had to be done manually and was a time consuming process. For this reason, we chose to investigate a random sample of 100 patients in this first exploratory study. This random sample of 100 patients was drawn out of the complete ROM-Phamous database, with the option ‘random sample of cases’ (under ‘select cases’) of IBM SPSS Statistics 20 [23].

### Measures

Demographic ROM-data were used, including gender, age and duration of illness. Demographic information of patients in the sample was compared to all patients in the ROM-database. Severity of psychiatric symptoms was assessed using the Positive and Negative Syndrome Scale Remission (PANSS-R) [24]. To assess positive symptoms, the items P1 ‘delusions’, P2 ‘conceptual disorganization’ and P3 ‘hallucinations’ were used and for negative symptoms, the items N1 ‘blunted affect’, N4 ‘passive/apathetic social withdrawal’ and N6 ‘lack of spontaneity and flow of conversation’ were used. Symptomatic remission is defined as ratings of mild (score 3) or less on all these items over a 6-month period [24]. Therefore, a score of 4 or higher on one or more of these items was defined as the presence of positive and/or negative symptoms. Assessment of psychosocial functioning, was based on the Dutch versions of the clinician-rated Health of the Nations Outcome Scale (HoNOS) and the self-report Manchester Short Assessment of Quality of Life (MANSA). More specifically, to measure problems in social relationships, item 9 ‘Problems with relationships’ of the HoNOS and item 12 ‘How satisfied are you with your social relationships?’ of the MANSA were used. To measure problems with daily activities and occupation, item 12 ‘Possibilities to use and improve skills: occupational and free time’ of the HoNOS and item 4 ‘How satisfied are you with your daily activities?’ of the MANSA were used. When the score on the HoNOS item was 3 or higher (indicating a problem that needs treatment) and/or the score on the MANSA item was 3 or lower (indicating dissatisfaction in this area), this was defined as a problem with social relationships/daily activities or occupation. To investigate cardiovascular risk factors, data from the physical examination were used, including measurement of weight, height and blood pressure, nicotine and cannabis use and additional laboratory tests. The modifiable risk factors for cardiovascular disease that were studied are overweight (body mass index higher than 25 kg/m^2^), diabetes mellitus (fasting plasma glucose levels >7.0 mmol/L), hypertension (blood pressure levels >140/90 mmHg (without diabetes) or >130/80 mmHg (with diabetes)) and dyslipidaemia (total cholesterol >5 mmol/L (without diabetes) or >4.5 mmol/L (with diabetes) and/or LDL-cholesterol >3 mmol/L (without diabetes) or >2.5 mmol/L (with diabetes)). These criteria were established by De Hert and others, in collaboration with the European Psychiatric Association [7], who have conducted extensive research on cardiovascular risk factors in people with severe mental illness. After calculating the prevalence of symptoms, psychosocial problems and cardiovascular risk factors with the ROM-data, the psychiatric treatment plans were investigated. It was scored whether the investigated problems were mentioned in the treatment plans: positive symptoms, negative symptoms, problems in social functioning, problems with daily activities, overweight, diabetes mellitus, hypertension, dyslipidaemia and smoking.

### Data analysis

The anonymized data were analysed by an independent researcher (MT). Descriptive statistics were performed in IBM SPSS Statistics 20 [23]. For each investigated problem area, patients were divided into four categories: 1) the problem was neither identified with ROM, nor reflected in the treatment plan, 2) the problem was not identified with ROM, but was reflected in the treatment plan, 3) the problem was identified with ROM, but not reflected in the treatment plan and 4) the problem was both identified with ROM and reflected in the treatment plan. Only patients of whom both the ROM-data and the psychiatric treatment plan were available were included in the analysis. Missing ROM-data were caused by patients who did not show up for appointments or refused to take part in (part of) the ROM-Phamous screening. Missing treatment plans were partly due to patients who stopped receiving care at our institution fairly soon after their ROM-screening and therefore did not have a new treatment plan. The reasons for the remaining missing treatment plans are unknown, but could be due to administrative problems or clinicians who failed to write treatment plans.

## Results

### Problems identified with ROM

Patient characteristics and the prevalence of the investigated problem areas, based on the ROM-data, are displayed in Table 1 for the selected sample. The total amounts of patients were different for each problem area, due to missing ROM-data. Most prevalent problems in our sample were positive symptoms, overweight, dyslipidaemia and smoking. No significant differences were found in gender distribution (*X*^2^ (1) = 0.026; *p* = 0.872), age (*t* = 0.979; *df* = 1137; *p* = 0.426) and duration of illness (*t* = −0.028; *df* = 896; *p* = 0.978) between the selected sample and the other patients in the database.

**Table 1:** Patient characteristics and prevalence of problems in the sample (*n* = 100), based on the ROM-data

Demographic information	
Male/female	63/37 (*n*)
Mean age (SD)	44.0 (10.3) (years)
Mean duration of illness (SD)	17.7 (9.0) (years)
DSM-IV classification	*n*
Schizophrenia	76
Schizo-affective disorder	13
Psychotic disorder NOS	11
Number of antipsychotic agents	*n*
No antipsychotic agents	10
One antipsychotic agent	75
Two or more antipsychotic agents	15
Symptoms and psychosocial problems	
Positive symptoms	37.2 % (32/86)
Negative symptoms	24.4 % (21/86)
Problems with social functioning	24.2 % (24/99)
Problems with daily activities/occupation	13.1 % (13/99)
Cardiovascular risk factors	
Overweight	66 % (62/94)
Diabetes mellitus type II	11.1 % (9/81)
Hypertension	10.9 % (10/92)
Dyslipidaemia	57.3 % (47/82)
Smoking	85.5 % (65/76)

### ROM and the treatment plan

**Fig. 1:**
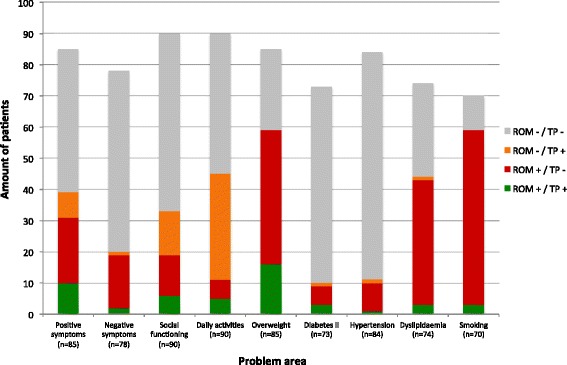
Problems detected with ROM and the treatment plan. This graph depicts whether problems were detected with ROM and whether they were reflected in the treatment plans of patients. The investigated problem areas are depicted on the x-as and the amount of patients is depicted on the y-as. Patients with incomplete information (missing ROM-data and/or missing treatment plans) are not shown in the graph. The *grey* part of the bars indicates the amount of patients of which a problem was neither detected with ROM, nor reflected in the treatment plan. The *orange* part of the bars indicates the amount of patients of which a problem was not detected with ROM, but was reflected in the treatment plan. The *red* part of the bars indicates the amount of patients of which a problem was detected with ROM, but was not reflected in the treatment plan. The *green* part of the bars indicates the amount of patients of which a problem was both detected with ROM and reflected in the treatment plan. (ROM = Routine Outcome Monitoring, TP = treatment plan, + = problem is detected/reflected, − = problem is not detected/reflected)

The time between the ROM-screening and the draft of the treatment plan ranged from 0 to 18 months (*mean* = 6.7; *sd* = 4.9). Figure 1 depicts whether problems were identified with ROM and whether they were reflected in the treatment plan. Only in a small amount of cases problems were both detected with ROM and reflected in the treatment plan (*mean* = 5; ranging from 1 patient to 16 patients between the different problem areas). In many cases, problems were detected with ROM, but not reflected in the treatment plan. This was especially striking for positive and negative symptoms, overweight, dyslipidaemia and smoking (*n* = 21; *n* = 17; *n* = 43; *n* = 40; *n* = 56, respectively). The opposite occurred as well, where problems were not detected with ROM, but were reflected in the treatment plans. This occurred most frequently in problems with social functioning and daily activities (*n* = 14; *n* = 34, respectively).

## Discussion

This study investigated to what extent ROM results of patients with a psychotic disorder translate to daily clinical practice. We found a substantial discrepancy between the ROM measurements and the treatment plans of patients, i.e. low rates of detection of symptoms, psychosocial problems and cardiovascular risk factors in the treatment plans, even though these problems were identified with ROM. The opposite occurred as well, where problems were reflected in the treatment plans but not identified with ROM. These exploratory results suggest that ROM and daily clinical practice are two separate processes, whereas ideally they should be integrated. An effort should be made to improve this integration, as this may help to provide patients with more adequate and customized care and simultaneously minimize under- and over-treatment.

### Implementation of ROM in clinical practice

Positive and negative symptoms, psychosocial problems and cardiovascular risk factors still occur relatively often in this population suffering from psychotic disorders. Our findings are in line with many other studies indicating that a high proportion of patients with psychotic disorders have little or no social contact or friends and experience difficulties getting involved in social activities or work [4]. Also, the prevalence of modifiable cardiovascular disease risk factors in our study is comparable with other studies in psychotic disorders [7, 12, 25]. Interestingly, whereas obesity was present in 66 % of patients, related problems such as diabetes mellitus and hypertension only occurred in a small subset of the sample. These low prevalences may indicate that these problems are already detected and treated relatively well, although these results should be replicated in a larger sample. Taken together, our ROM-system is sensitive for the detection of relevant problems in patients with a psychotic disorder.

However, the most striking finding is that clinicians do not adjust the treatment according to the ROM results. Our findings illustrate that ROM is not implemented to its full benefit in the healthcare provided for these patients. These findings also underline the notion that merely introducing a ROM-system in mental health care is not enough [26, 27], as research shows that few clinicians use the outcome of ROM in their day-to-day work [15, 16]. There appear to be barriers and challenges in the use of routine outcome data [28], including the fact that clinicians are often overscheduled and experience time pressure [28, 29]. This was also reflected in the substantial time gap between the ROM-screening and the treatment plans of patients, found in the present study.

Clearly, the implementation of ROM results in the treatment of patients must be improved. A first step should be to have the interpretation of ROM-results and the updating of the treatment plan within a limited time frame. This would help to reduce the long time between the ROM-screening and the draft of the treatment plan that we found in our study. Also, to improve the standard of care, the decision making process of clinicians could be made more explicit and transparent. To this end, all identified problems must be described in the treatment plan. Next, considerations should be described about whether or not treatment will be given. This will help to avoid certain problems to be discussed repeatedly and, more importantly, will avoid that problems are not taken into consideration. In addition, it should be clearly stated when treatment is not given and whether this is because the available treatment options did not work or whether it is due to lack of availability of care. The first issue should be addressed with treatment research and strict adherence to treatment guidelines, whereas the latter should be addressed with health care policy adjustments.

### The focus of treatment

An interesting result of our study was the finding that in some cases problems were reflected in the treatment plan, but were not identified with ROM. This was mostly seen in problems with social functioning and problems with daily activities. Presumably, our ROM measures did not pick up these signals or patients may not have reported these problems due to lack of insight or an adjustment of expectations to their chronic situation. Unexpectedly, we found this discrepancy for positive symptoms as well. Possibly, positive symptoms are still mentioned as treatment goals, even when symptoms do not interfere with daily life functioning anymore and therefore are not detected with ROM. In general, it is possible that positive symptoms and psychosocial problems already receive much attention in clinical practice. This could be because these problems usually stand out and are difficult to overlook. Apparently, the clinician’s focus of treatment seems to be directed most strongly on psychotic symptoms and psychosocial symptoms and less on negative symptoms and physical health.

### Strengths and limitations

Strength of the study is the broad scope, as multiple areas of functioning were investigated with ROM. Moreover, the sample is an epidemiologically representative sample from the majority of patients with schizophrenia in the province of Groningen, the Netherlands. Also, the sample is representative for all patients in the ROM database. There are some limitations of the study. Firstly, our data was only retrieved from clinical records and we did not explicitly ask clinicians about their decision making process. It is therefore possible that certain problems that were identified with ROM, were not reflected in the treatment plan for specific reasons we are not aware of. Lastly, the data was collected and scored by one independent researcher. In future studies, data collection should be done by at least two independent researchers to improve reliability.

### Future research

This study prompts our mental health care organization as well as others to actively investigate the current state of affairs. We expect to see improvements in the implementation of ROM within the next years. All stakeholders should aim to bridge the gap between ROM and the treatment of patients as this may help to improve the quality of care, especially in patients needing long term care. To achieve this, it would be helpful to develop software applications that can reduce time burdens and make ROM outcome assessment more user-friendly for clinicians and patients [28]. We are currently working on a tool that helps translate and interpret ROM outcome and in addition offers suggestions for treatment based on the most recent treatment guidelines. We hope this can further improve the integration between ROM and treatment.

## Conclusions

Our study investigated to what extent ROM results of patients with a psychotic disorder translate to daily clinical practice. We found a substantial discrepancy between the ROM measurements and the treatment plans of patients. These exploratory results suggest that the connection between ROM and the treatment that patients receive is far from optimal yet. Strong efforts should be made to integrate routine outcome monitoring and consequent treatment activities. Such integration may help to provide patients with the most adequate and customized care and simultaneously minimize under- and over-treatment.

## Abbreviations

ROM: routine outcome monitoring
ROM-Phamous: routine outcome monitoring pharmacotherapy monitoring and outcome survey
UMCG: University Medical Center Groningen
EPF: electronic patient file
PANSS-R: positive and negative syndrome scale remission
HoNOS: health of the nations outcome scale
MANSA: Manchester short assessment of quality of life
SRA-34: subjects’ response to antipsychotics
